# Intrathecal Morphine and Post-Operative Pain Relief in Robotic Surgeries: A Systematic Review and Meta-Analysis

**DOI:** 10.3390/jcm13010137

**Published:** 2023-12-26

**Authors:** Zi Heng Tee, Erica Ho Ching Tsoi, Quinston Lee, Yen Sin Wong, Arron Gibson, Niamh Parsons, Shafaque Shaikh, Patrice Forget

**Affiliations:** 1 School of Medicine, Medical Sciences and Nutrition, University of Aberdeen, Aberdeen AB25 2ZD, UKshafaque.shaikh@nhs.scot (S.S.); patrice.forget@abdn.ac.uk (P.F.); 2 Department of Surgery, Aberdeen Royal Infirmary, NHS Grampian, Aberdeen AB25 2ZD, UK; 3 Epidemiology Group, Institute of Applied Health Sciences, School of Medicine, Medical Sciences and Nutrition, University of Aberdeen, Aberdeen AB25 2ZD, UK; 4 Department of Anaesthesia, Aberdeen Royal Infirmary, NHS Grampian, Aberdeen AB25 2ZD, UK; 5 Pain and Opioids after Surgery (PANDOS) Research Group, European Society of Anaesthesiology and Intensive Care, B-1000 Brussels, Belgium

**Keywords:** intrathecal morphine, post-operative outcomes, robotic surgery

## Abstract

Despite the potential benefits of intrathecal morphine (ITM), the precise role and dosing of ITM in robotic assisted surgery (RAS) remains unclear. This systematic review explores real-world evidence to evaluate the efficacy and outcomes of ITM in patients undergoing RAS. In accordance with PRISMA guidelines, a comprehensive search was conducted on four databases: MEDLINE, Embase, Cochrane Library and APA PsycInfo. Primary outcomes included pain scores at rest and on exertion at 24- and 48-h time intervals, and secondary outcomes aimed to explore the side effects of ITM. A meta-analysis was conducted to determine mean differences. A risk of bias assessment was conducted via the Cochrane Risk of Bias 2 tool. A total of 9 RCTs involving 619 patients were included in this review, of which 298 patients were administered ITM. Significant pain score reductions were observed both at rest (MD = −27.15; 95% CI [−43.97, −10.33]; I^2^ = 95%; *p* = 0.002) and on exertion (MD = −25.88; 95% CI [−37.03, −14.72]; I^2^ = 79%; *p* = 0.0003) 24 h postoperatively in the ITM groups, accompanied by a notable decrease in postoperative IV morphine equivalent consumption at 24 h (MD = −20.13; 95% CI [−30.74, −9.52]; I^2^ = 77%; *p* = 0.0002). ITM improved pain scores both at rest and on exertion at 24 and 48 h intervals, concurrently reducing the need for postoperative opioid consumption, but at the cost of an increased incidence of adverse events.

## 1. Introduction

Current postoperative pain management strategies like opioids carry risks of adverse effects and inadequate pain relief. This can lead to delayed mobilisation, chronic pain development, and extended hospital stays [1].

Morphine is an opioid administered for acute and chronic pain conditions [2]. As an affordable, effective, and well-tolerated analgesia, morphine is often used for postoperative pain management, either via oral (PO), intravenous (IV), subcutaneous (SC), intramuscular (IM) or intrathecal (commonly described as spinal) routes. However, administration of PO and IV morphine remains challenging since, sometimes, a larger amount of morphine is required to achieve an analgesic effect. Consequently, patients are at a greater risk of side effects such as nausea and vomiting. SC and IM morphine routes have been shown to have a time delay in analgesic effects in the immediate postoperative period [3].

Delivered via the intrathecal or subarachnoid space in the lower lumbar levels, intrathecal morphine (ITM) directly interacts with opioid receptors (mu, delta and kappa) and ion channels located in the dorsal horn of the spinal cord [2,4]. This leads to an overall decrease in the release of excitatory transmitters and an increase in inhibitory transmitters within pain pathways. Therefore, nociceptive pain signals are significantly reduced [5]. Moreover, ITM administration in the cerebrospinal fluid allows for bypass of first-pass metabolism and the blood–brain barrier [6]. As a result, analgesic effects from ITM can be achieved at a lower dose with fewer adverse effects, together with a longer duration of action that lasts up to 20 h [2]. Correspondingly, ITM is an increasingly accepted form of perioperative and postoperative analgesia.

First described by Wang et al. in 1979, ITM was successfully utilised in patients with genitourinary tract malignancies [7]. Subsequently, ITM is indicated for pain management in various surgeries [8].

Despite present pain management guidelines surrounding the risk/benefit ratio of ITM, its efficacy and safety in robotic-assisted procedures remain unclear. Prior studies have had small sample sizes and inconsistent results on optimal dosing to balance analgesia and side effects [9,10]. Given that ITM has the potential to improve postoperative RAS outcomes, we aimed to systematically review the available evidence on ITM for postoperative pain management in robotic surgery. Our objectives were to assess its effects on pain scores, opioid use and adverse events compared to other analgesia techniques.

## 2. Materials and Methods

This systematic review was performed in accordance with the Preferred Reporting Items for Systematic Review and Meta-analysis (PRISMA-P) guidelines and was registered in the International Prospective Register of Systematic Reviews (PROSPERO) (CRD42023405398) [11].

We searched Pubmed, Embase, Cochrane Library and PsyInfo using defined search terms for relevant randomised controlled trials. The search was last performed on 11 March 2023. We utilised the online platform Rayyan QCRI to perform deduplication and screening of papers via title and abstract independently [12]. A full-text review was subsequently conducted for final inclusion into our systematic review while adhering to our selection criteria. Two authors (Z.H.T. and E.T.) independently screened records and extracted data on study characteristics, patient demographics, ITM dosing, spinal anaesthesia details, control analgesia and key outcomes. Any conflicts were resolved by consensus or appeal to the senior author (P.F.).

### 2.1. Selection Criteria

Utilising the Populations, Interventions, Comparators and Outcomes (PICO) framework from the Cochrane Handbook of Systematic Reviews, we selected studies based on the following inclusion criteria: (a) RCTs/ongoing RCTs of adult patients (≥18 years old) undergoing robotic/robot-assisted surgeries with administration of ITM, (b) comparison of any other analgesia/anaesthetic techniques apart from ITM or placebo/saline control groups, and (c) inclusion of pain scores, opioid consumption by patients and any adverse drug reactions [13].

Studies with paediatric patients were excluded. Studies reporting minimally invasive surgeries were excluded. Non-english studies, non-RCTs, as well as animal studies were excluded. Our inclusion and exclusion criteria can be found in the PICO table (Table 1).

### 2.2. Data Extraction and Quality Assessment

A data sheet was utilised to extract relevant data from our included studies. Primary outcomes analysed were pain scores on a 0–100 VAS at 24 and 48 h and opioid consumption at 24 h in morphine equivalents. Secondary outcomes analysed were adverse event rates at a 24 h time interval.

We defined the primary outcome of interest in accordance with the British Pain Society and Faculty of Pain Medicine guidelines [14]. These include the verbal rating scale, a visual analogue scale or a numerical rating scale. A questionnaire developed by the British Pain Society requires patients to score their pain on a scale of 1–10 to classify the intensity of their pain. Studies that reported Numeric Rating Scale (NRS) pain scores from 11 (0–10) or 10 (1–10) point scales were transposed to a 0 (no pain)–100 (worst imaginable pain) Visual Analogue Scale (VAS) scale. For studies that presented pain scores categorised as mild, moderate and severe, the mean score within each category was extracted and transposed to the VAS scale.

The PROSPECT methodology suggests a mean difference of 10 mm on a VAS scale should be considered a statistically significant effect [15]. As such, a statistically significant difference in effect size of >10.0 was considered to be clinically relevant in the meta-analysis.

All postoperative opioid and rescue analgesic consumption at 24 h were converted to IV morphine equivalent, where the dose equivalent is defined as follows: 100 µg IV fentanyl = 100 mg IV tramadol = 100 mg IV pethidine = 10 mg IV morphine [16,17].

The secondary outcome aims to explore side effects such as nausea, vomiting, pruritus, urinary retention, constipation and respiratory depression at 24 h, postoperatively. Nausea is defined as an unpleasant sensation of needing to vomit, which can be accompanied by autonomic symptoms such as salivation, pallor, tachycardia and hidrosis [18]. Vomiting is the ejection of the contents from the stomach through the mouth [19]. Pruritus is defined as severe itching of the skin coupled with the desire to scratch [20]. Urinary retention is defined as the inability to voluntarily pass an adequate volume of urine [21]. Constipation is defined as a reduction in the frequency or difficulty of evacuating stools [22]. Opioid-induced respiratory depression is defined as SaO_2_ <94% and/or PaCO_2_ >6 kPa, or a respiratory rate <6 breaths per min [23].

### 2.3. Risk of Bias Assessment

Studies were evaluated for a risk of bias using the Cochrane Risk of Bias 2 (RoB2) tool across 5 domains [24]. Each study was assessed twice by two independent reviewers. Any discrepancies were resolved through discussion with the senior author. Each domain was assessed as “high risk”, “some concerns” or “low risk” with the inclusion of an overall risk of bias.

### 2.4. Statistical Analysis

Data were presented as percentages with raw values or means ± the standard deviation unless otherwise indicated. For studies that did not present in the above format, we attempted to contact the corresponding authors for access to the relevant data or complete dataset. If there were no responses, data presented as the median (IQR) was approximated as the mean ± the standard deviation, where the median was estimated as the mean and the standard deviation as the IQR divided by 1.35.

Meta-analyses of all listed outcomes were conducted on Review Manager [RevMan Version 5.4] software (Cochrane Collaboration, Copenhagen, Denmark). Due to high heterogeneity, the random effects model was used for a pooled meta-analysis of continuous variables with the effect sizes, 95% confidence interval and *p* values calculated to signify statistical significance. Heterogeneity was assessed with I^2^ values and Cochran’s Q test, where <50% represented low heterogeneity and ≥50% represented substantial heterogeneity [25].

Sub-analyses were conducted based on the surgical procedure according to primary and secondary outcomes. Sensitivity analyses were performed, excluding individual studies and controlling for ITM-only spinal anaesthesia. Publication bias was visually interpreted via funnel plots for each time point (24 and 48 h) for pain scores at rest and on exertion [26].

## 3. Results

### 3.1. Search Results

**Figure 1:**
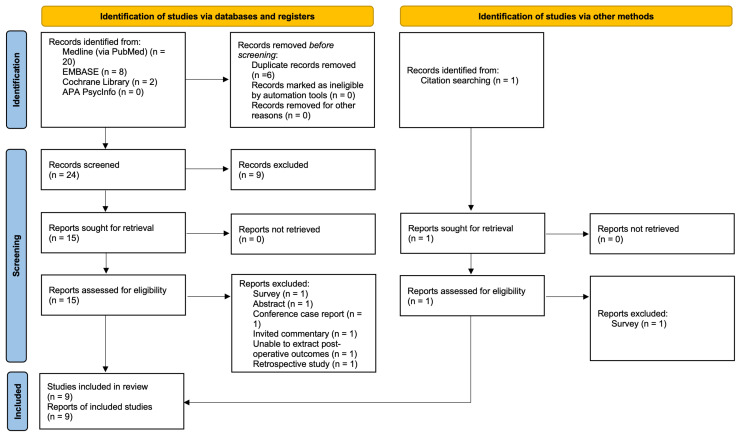
PRISMA flow diagram of included studies [35].

A total of 31 studies were identified, of which 16 studies were screened for full-text review after removal of duplicates and exclusion via title and abstract. Of the 16 studies, 7 studies were excluded. Reasons for exclusion are as follows: surveys, conference case reports, invited commentary or unable to extract postoperative outcomes. Quality assessment was performed on all nine included studies using the Cochrane Rob2 tool, and no study was excluded after quality assessment [9,27,28,29,30,31,32,33,34] (Figure 1).

### 3.2. Study Characteristics

All included studies were published between 2013 and 2023 and were from six different countries (Israel, Italy, Republic of Korea, The Netherlands, Sweden and USA). Types of robotic procedures include prostatectomy, nephrectomy, hysterectomy, sacrocolpopexy and coronary artery bypass. A total of 619 patients were included, of which 298 patients were in the ITM group and 321 patients were in the control group.

Within the included studies, the dose of ITM ranged from 0.10 mg to 0.50 mg. The use of spinal anaesthesia was reported across five studies, where all studies reported the use of bupivacaine except Segal et al., 2013, who reported the use of fentanyl [32]. The types of controls ranged from sham procedure, normal saline and other anaesthetic agents such as IV fentanyl and propofol, IV morphine, IV-PCA (fentanyl, ketorolac and ramosetron), IV tramadol, TAP block with ropivacaine, and oral oxycodone. The study characteristics are summarised in Table 2.

### 3.3. Primary Outcomes

#### 3.3.1. Pain Scale Methods

All nine studies reported pain scores using either the Numeric Rating Scale (NRS) or Visual Analogue Score (VAS). The majority of the studies reported both pain at rest and on exertion, except Koning et al., 2020 [9], which reported pain on exertion and Chae et al., 2022 [28], that reported pain at rest. Pain scores at rest or on exertion were not specified in Engström et al., 2023 [30]. All studies specified time intervals at which the pain scores were measured except Engström et al., 2023 [30].

#### 3.3.2. Pain Scores at Rest after Postoperative ITM at 24 h

Five studies reported pain scores at rest in the ITM and control groups at 24 h time intervals [27,29,32,33,34] (Table 3). The meta-analysis found ITM significantly reduced pain scores at rest (MD = −27.15; 95% CI [−43.97, −10.33]) compared to controls. Statistical heterogeneity is considerable at I^2^ = 95%, and Cochran’s Q test revealed *p* = 0.002.

**Figure 2:**
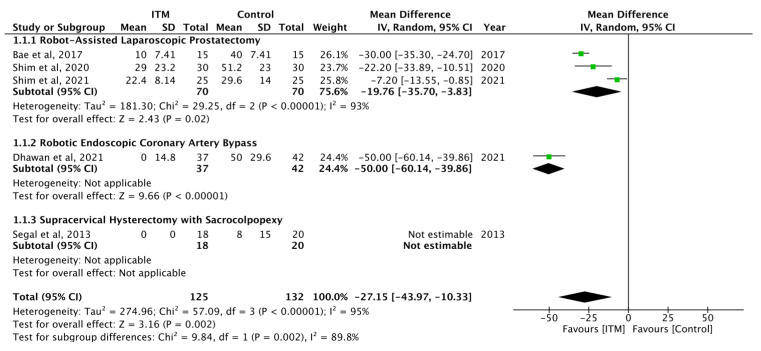
Pain scores at rest after postoperative ITM at 24 h [27,29,32,33,34].

In three studies reporting robotic-assisted laparoscopic prostatectomy (RALP), ITM also decreased pain scores at rest at 24 h (MD = −19.76; 95% CI [−35.70, −3.83]). Statistical heterogeneity is considerable at I^2^ = 93%, and Cochran’s Q test revealed *p* = 0.02 (Figure 2).

#### 3.3.3. Pain Scores on Exertion after Postoperative ITM at 24 h

**Figure 3:**
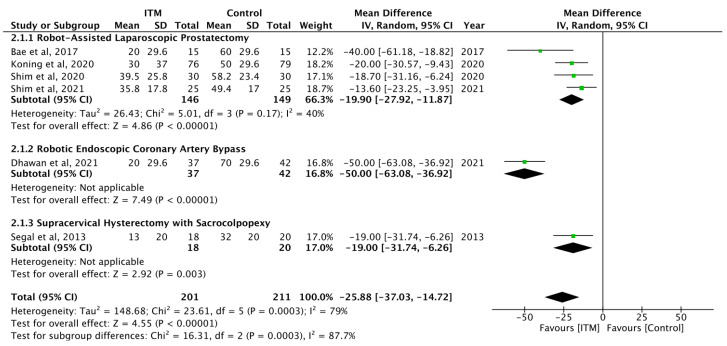
Pain scores on exertion after postoperative ITM at 24 h [9,27,29,32,33,34].

ITM significantly lowered exertional pain scores at 24 h in six studies (MD = −25.88; 95% CI [−37.03, −14.72]) and specifically in robotic prostatectomy patients (MD = −19.90; 95% CI [−27.92, −11.87]) [9,27,29,32,33,34] (Figure 3).

#### 3.3.4. Pain Scores on Rest and after Exertion after Postoperative ITM at 48 h

A total of four studies reported pain scores 48 h postoperatively [27,28,29,32]. Of which, two studies reported lower pain scores on both categories in the ITM group. Only Segal et al., 2013 [32], reported higher pain scores in the ITM group compared to the control group at rest and on exertion. Pain scores at rest and on exertion were not specified in Chae et al., 2022 [28], and pain scores on exertion were not specified in Dhawan et al., 2021 [29] (Table 3).

#### 3.3.5. Postoperative Consumption of Equivalent IV Morphine Consumption at 24 h

**Figure 4:**
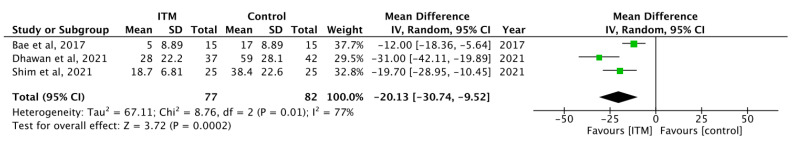
Postoperative consumption of equivalent IV morphine consumption at 24 h [27,29,34].

A total of four studies were observed to have a reduction in postoperative IV morphine equivalent consumption at 24 h in the ITM groups [27,29,32,34] (Table 3). Except for Bae et al. [27] due to missing data, the meta-analysis of three studies found that ITM reduced 24 h IV morphine equivalent consumption (MD = −20.13; 95% CI [−30.74, −9.52]). Statistical heterogeneity is considerable at I^2^ = 77%, and Cochran’s Q test revealed *p* = 0.0002 (Figure 4).

### 3.4. Secondary Outcomes

#### 3.4.1. Nausea and Vomiting

A total of four studies reported an increased incidence of nausea in the ITM groups, ranging from 22.2% to 36.4% [29,31,33,34]. Use of antiemetics as prophylaxis and/or treatment was also reported across eight studies [9,30,31,32,33,34]. Bae et al., 2017 [27], reported no difference in the incidence of nausea, though two patients in the control group required 10 mg of metoclopramide. Prophylaxis of nausea and vomiting included ondansetron, dehydrobenzperidol, ramosetron and betamethasone, while treatment included ondansetron, dehydrobenzperidol and metoclopramide. Likewise, four studies reported an increased incidence of vomiting in the ITM groups, ranging from 0% to 20% [27,29,33,34]. More information can be found in Table S1.

**Figure 5:**
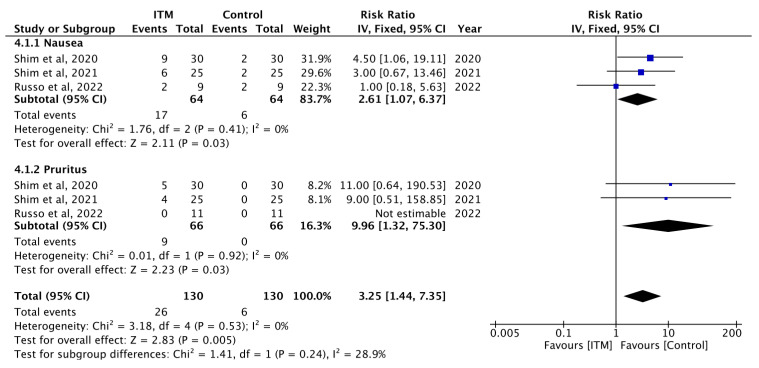
Secondary outcomes of included studies at 24 h [31,33,34].

For studies that reported events of nausea at 24 h time intervals, ITM increased the risks of nausea (RR = 2.61; 95% CI [1.07, 6.37]). Statistical heterogeneity is low at I^2^ = 0%, with Cochran’s Q test revealing *p* = 0.03 (Figure 5).

#### 3.4.2. Pruritus

A total of five studies reported an increased incidence of pruritus in the ITM groups, ranging from 0% to 60% [27,29,32,33,34] (Table S1). Bae et al., 2017 [27], reported a substantial difference with 14% of patients experiencing pruritus in the control group, whereas 60% of patients experienced pruritus in the ITM group. For studies that reported the incidence of pruritus at 24 h time intervals, ITM increased the risks of pruritus (RR = 9.96; 95% CI [1.32, 75.30]). Statistical heterogeneity is low at I^2^ = 0%, with Cochran’s Q test yielding *p* = 0.03 (Figure 5).

#### 3.4.3. Urinary Retention

Engstrom et al., 2023 [30], was the only study to note a higher incidence of urinary retention (6.52%, n = 3) in the ITM group as opposed to the control group (4.17%, n = 2); however, it is not possible to determine the statistical significance of this finding due to the small study numbers. Notably, Dhawan et al., 2021 [29], documented a urinary retention rate of 2.7% (n = 1) in the control group, whereas no patients in the ITM group experienced this issue. Segal et al., 2013 [32], reported no difference in urinary retention in either the control or the ITM groups (Table S1).

#### 3.4.4. Respiratory Depression

Amongst the studies which reported the incidence of respiratory depression, five studies found no incidence of respiratory depression events in either the control or the ITM groups [27,29,30,31,34] (Table S1).

### 3.5. Quality Assessment and Risk of Bias

**Figure 6:**
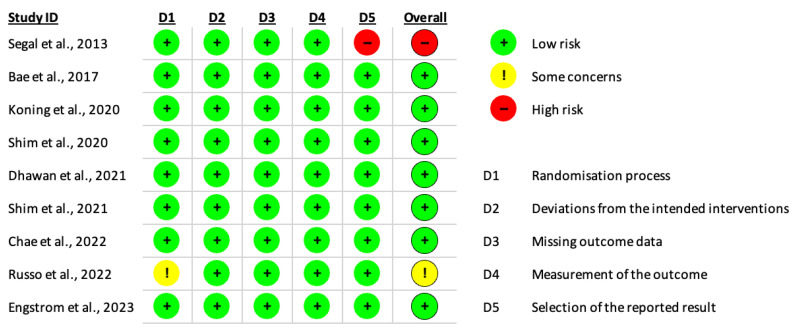
Cochrane Rob2 risk of bias assessment summary [9,27,28,29,30,31,32,33,34].

Using the Cochrane Rob2 Tool, studies were investigated for bias by two independent researchers [24]. Any discrepancies were resolved through discussion. Bias was evaluated under five domains and was assessed to have a low risk, some concerns or a high risk of bias. A summary of our risk of bias assessment can be found in Figure 6.

Russo et al., 2022 [31], did not report their randomisation process and did not specify if participants, clinicians and researchers were blinded during the trial. However, there were no significant differences in the baseline characteristics between the intervention and control groups, thus we evaluated the study to be of some concerns in bias.

Pain scores were measured every 30 min until discharge in Segal et al., 2013 [32]. However, pain scores were only reported in the recovery unit, postoperative day 1 and postoperative day 2. This highlights a high risk of bias as the reported results could have been selected on the basis of results.

### 3.6. Test for Statistical Heterogeneity

Multiple instances were detected where the removal of one study changed the results of an analysis from statistically insignificant to statistically significant for primary outcomes exploring pain scores on rest at 24 h time intervals and secondary outcomes, with the exception of Shim et al., 2021 [34], and Russo et al., 2022 [31], respectively. The sensitivity analysis of the studies did not find any evidence of a significant change in the analysis for reported primary outcomes with regards to pain scores on exertion at 24 h time intervals. Exclusion of studies with a high risk of bias did not find any significant changes in the reported primary outcomes.

Sensitivity analysis was attempted after excluding all studies not including “basic analgesics” in both groups, where “basic analgesics” is defined as the regular or documented use of paracetamol and any NSAIDs not only as rescue analgesia but as regularly/systematically prescribed or documented/quantified in the studies. Due to missing data in Russo et al., 2022 [31], three studies were identified, and sensitivity analysis was performed on pain scores on exertion after postoperative ITM at 24 h [9,33,34]. Revised sensitivity analysis revealed a significant improvement in pain scores in the ITM group (MD = −17.05; 95% CI [−23.24, −10.86]). Statistical heterogeneity is low at I^2^ = 0%, with Cochran’s Q test yielding *p* < 0.00001 (Figure S1).

### 3.7. Publication Bias

The funnel plot was based on the chosen outcome with the highest number of studies, i.e., pain scores on exertion after postoperative ITM at 24 h. Visual analysis of the funnel plot did not allow us to confirm any risk of publication bias (Figure S2).

## 4. Discussion

Whilst the use of other pre-emptive, systemic and regional analgesic techniques proves effective in pain management, ITM effectively reduced pain scores and opioid use in the first 24 h after various robotic surgeries, aligning with its pharmacological effects. [36]. Compared with the control groups, our study reflects significant improvements in pain scores at rest (MD = −27.15; 95% CI [−43.97, −10.33]; I^2^ = 95%; *p* = 0.002) and pain scores on exertion (MD = −25.88; 95% CI [−37.03, −14.72]; I^2^ = 79%; *p* = 0.0003) in the ITM groups at 24 h, postoperatively. This is reflected in the reduced postoperative opioid consumption at 24 h in the ITM group as well (MD = −20.13; 95% CI [−30.74, −9.52]; I^2^ = 77%; *p* = 0.0002).

However, benefits must be weighed against increased postoperative side effects. For the purposes of this review, the side effects that were noted in the selected trials include nausea, vomiting, pruritus and respiratory depression. Based on our results, there is an increased incidence of nausea (MD = 2.61; 95% CI [1.07, 6.37], I^2^ = 0%, *p* = 0.03) and pruritus (MD = 9.96; 95% CI [1.32, 75.30], I^2^ = 0%, *p* = 0.03) at 24 h time intervals. Although respiratory depression is the most serious adverse side effect of ITM, there was no incidence of respiratory depression events found in either the ITM or the control groups [27,29,30,32,35]. This may be due to the dose of ITM utilised ranging from 0.10 mg to 0.50 mg, highlighting the dose dependent relationship between ITM and respiratory depression. Though greater analgesic effects can be achieved with higher doses of ITM (>500 µg), it is also associated with significant adverse events, most notably respiratory depression [37,38,39]. The insufficient studies available meant that we were unable to conduct a meaningful analysis of the dose-dependent relationship between ITM and different robotic procedures.

Due to the heterogeneity of the reported secondary outcomes, a meaningful comparison could only be made for papers that reported side effects post 24 h via meta-analysis [31,33,34]. Another limitation in our data collection is the documentation of nausea and vomiting as a collective measure rather than individual occurrences in Russo et al., 2022 [31], of which we assumed all patients to have nausea. Most studies only included patients with an ASA status of 1–2, which makes the incidence of other well-known ITM side effects such as haemodynamic hypotension, headaches and seizures difficult to determine, especially in high-risk patients [3]. The use of antiemetics postoperatively such as metoclopramide and ondansetron in some studies can mask the incidence of postoperative nausea and vomiting, which may be underreported [9,27,31].

Only four studies reported the use of regular basic analgesics in both ITM and control groups as per the PROSPECT guideline methodology [9,31,33,34]. Alignment of routine clinical practice allows an accurate assessment of ITM and the ability to determine if ITM provides additional pain relief on top of basic analgesics with or without locoregional analgesia in the overall pain management strategy. As a result, the sensitivity analysis is limited to only three studies reporting pain scores on exertion after postoperative administration of ITM at 24 h (MD = −17.05; 95% CI [−23.24, −10.86]; I^2^ = 0%; *p* < 0.00001). A recommendation for future research is to conduct studies with basic analgesics in both arms for pain scores at rest and on exertion at different time intervals.

Robotic surgery has been an increasingly popular surgical approach since its establishment in 1979. Since then, over 10 million procedures have been performed worldwide, with an increase from 1.8% to 15.1% across all general surgical procedures in Michigan alone from 2012 to 2018 [40,41]. However, the advent of RAS remains relatively new, with a lack of clear established guidelines in the context of postoperative pain management. As a derivative of laparoscopic surgery, postoperative pain in RAS is associated with several underlying mechanisms: incisional port site pain, pneumoperitoneum and referred pain [42]. CO_2_ insufflation in the peritoneum leads to shearing of blood vessels, traction on nerve endings and release of inflammatory mediators. Residual gas within the peritoneal cavity induces diaphragmatic stretching and irritation of the phrenic nerve, resulting in shoulder, abdominal or back pain [43]. Furthermore, patients undergoing RAS are required to be positioned at a steeper angle, i.e., the Trendelenburg position, which increases the risk of positional injuries and synergistic complications of pneumoperitoneum and referred pain. However, postoperative visceral pain following RAS is often short-lived and typically resolves within 24 h. When combined with the extended analgesic benefits of ITM lasting up to 20 h, ITM may prove to be a valuable postoperative analgesic intervention for RAS.

It should be noted that most included studies remain heterogeneous with different follow-up time periods and reported pain scales. Limitations include clinical and moderate heterogeneity between studies, small sample sizes and a lack of long of long-term follow-ups. As a result, our statistical analysis was limited to studies that reported pain outcomes at 24 h and 48 h, postoperatively. Moreover, most studies did not adhere to definitions for pain scales according to ACTTION, where traditional pain scales such as NRS or VAS pain scales are defined as 0–10 and 0–100, respectively [44]. To have a meaningful analysis of pain scores from our included studies, we adapted pain scales to the VAS pain scale for standardisation. Furthermore, limited studies for postoperative equivalent IV morphine consumption at a 24 h time interval prevented us from making a meaningful analysis among different robotic procedures. Subgroup analysis for primary outcomes could only be localised to RALP studies due to the lack of studies in other robotic procedures. Due to the paucity of studies that included basic analgesics with or without locoregional anaesthesia in both ITM and control groups, the sensitivity analysis was also limited to pain scores on exertion after postoperative ITM at 24 h.

## 5. Conclusions

Our study demonstrates that ITM improved pain scores at rest and on exertion at 24 h and 48 h time intervals with reduced postoperative opioid equivalent consumption. However, pain management in most studies was not PROSPECT compliant, in particular to the systematic use of non-opioids. To accurately assess the true effectiveness of ITM in RAS, future large RCTs should compare ITM to alternative active comparators, intravenous morphine bolus administered under general anaesthesia, or combinations with locoregional analgesics in specific robotic procedures with the standardisation of protocols and outcomes. Cost effectiveness studies are needed to determine whether reduced opioid use offsets increased risks of ITM side effects. Overall, ITM holds promise as an opioid-reducing adjuvant, but optimal patient selection and dosing requires further research.

### Future Development/Gaps

This study highlights the need for further research into the prospects of employing alternative perioperative analgesia protocols in specific RAS procedures to allow for the standardisation of analgesic techniques. Furthermore, a standardised pain evaluation tool also needs to be developed to facilitate a better understanding of the variation of the perception of pain in different patients.

## Figures and Tables

**Table 1:** Inclusion and exclusion criteria for selection criteria of studies.

	Inclusion Criteria	Exclusion Criteria
Population	Adult patients (≥18 years old)	Paediatric patients
	Undergoing robot-assisted/robotic surgeries	Any other surgical approach
Intervention	Administration of ITM in patients who have undergone robot-assisted/robotic surgery	Any other intervention
Comparisons	Any other analgesia/anaesthetic techniques apart from ITM in postoperative patients	No comparison
	Placebo/saline control groups in postoperative patients	
Outcomes	Pain scores: verbal rating scale, visual analogue scale and numerical rating scale	No data on pain score
	Opioid consumption by patients	
	Adverse drug reactions: nausea, vomiting, pruritus, urinary retention, constipation and respiratory depression	
Study Type	- RCTs - Ongoing RCTs	- Non-RCTs - Unrelated to research question - Animal studies - Untranslated foreign articles - No results

**Table 2:** Overview of included studies.

Author(s)	Country	Publication Year	Study Period	Type of Surgery	ITM Group		Spinal Anaesthesia	Control (n)	Type of Control
					n	Dose (mg)			
Segal et al., 2013 [32]	Israel	2013	2011–2012	Robotic sacrocolpopexy ± subtotal hysterectomy	18	0.15–0.50 ^†^	15 mcg fentanyl	20	1–2 mcg/kg IV fentanyl and 1–3 mg/kg propofol
Bae et al., 2017 [27]	Korea	2017	2013–2014	RALP	15	0.30	NR	15	Saline with 1 mcg/mL IV morphine
Koning et al., 2020 [9]	Netherlands	2020	2016–v2018	RALP	76	0.24/0.30	12.5/10 mg isobaric bupivacaine	79	Sham procedure with 0.1 mg/kg morphine intraoperatively
Shim #1 et al., 2020 [33]	Korea	2020	May 2020–July 2020	RALP	30	0.20	7.5 mg bupivacaine	30	1000 mcg fentanyl 90 mg ketorolac 0.3 mg ramosetron
Dhawan et al., 2021 [29]	USA	2021	2018–2020	Robotic endoscopic CABG	37	0.42 ±0.07 *	NR	42	1mL saline
Shim #2 et al., 2021 [34]	Korea	2021	Oct 2019–Dec 2019	RALP	25	0.20	7.5 mg bupivacaine	25	1000 mcg fentanyl 90 mg ketorolac 0.3 mg ramosetron
Chae et al., 2022 [28]	Korea	2022	2020–2021	Robot-assisted laparoscopic partial nephrectomy	40	0.20	NR	40	0.5 mL saline
Russo et al., 2022 [31]	Italy	2022	2020–2021	RALP	11	0.15	NR	22	400 mg IV tramadol in 48 mL of 0.9% NaCl solution (n = 11) TAP block with 20 mL 0.2% ropivacaine (n = 11)
Engström et al., 2023 [30]	Sweden	2023	2021–2022	Robotic-assisted laparoscopic hysterectomy	46	0.10	15 mg bupivacaine	48	10 mg oxycodone oral

Legend: CABG Coronary Artery Bypass Graft, ITM Intrathecal Morphine, NR not reported, RALP Robot-Assisted Laparoscopic Prostatectomy, TAP Transversus Abdominal Plane, USA United States of America. Continuous data are presented as absolute values unless otherwise stated. ^*^ Value represented as mean ± standard deviation. ^†^ Value represented as range only.

**Table 3:** Primary outcomes of included studies.

	ITM Group						Control					
Author(s)	Type of Intervention	24 h (Converted)		48 h (Converted)		Postoperative IV Morphine Equivalent at 24 h (mg)	Type of Control	24 h (Converted)		48 h (Converted)		Postoperative IV Morphine Equivalent at 24 h (mg)
		At rest	On Exertion	At Rest	On Exertion			At Rest	On Exertion	At Rest	On Exertion	
Segal et al., 2013 [32]	15 mcg fentanyl + 0.15–0.5 mg ITM + 1–2 mcg/kg IV fentanyl + 1–3 mg/kg propofol	0	13 ±20.0	3 ±0	19 ±30.0	0.33 ^*^	1–2 mcg/kg IV fentanyl + 1–3 mg/kg propofol	8 ±15.0	32 ±20.0	0	15 ±20.0	7.59 ^*^
Bae et al., 2017 [27]	0.30 mg ITM + 100 mg morphine + IV-PCA	10 ±7.41	20 ±29.6	5 ±7.41	30 ±25.2	5 ±8.89	100 mg morphine + normal saline (IV-PCA)	40 ±7.41	60 ±29.6	20 ±18.7	15 ±20.0	17 ±8.89
Koning et al., 2020 [9]	0.30 mg/5 mL ITM + 12.5 mg bupivacaine OR 0.24 mg/4 mL ITM + 10 mg bupivacaine	NR	30 ±37.0	NR		NR	Sham procedure with 0.1 mg/kg morphine	NR	50 ±29.6	NR		NR
Shim #1 et al., 2020 [33]	0.20 mg ITM + 7.5 mg bupivacaine + IV-PCA	29 ±23.2	39.5 ±25.8	NR		NR	1000 mcg fentanyl + 90 mg ketorolac + 0.3 mg ramosetron (IV-PCA)	59.3 ±23.8	71 ±21.4	NR		NR
Dhawan et al., 2021 [29]	5 mcg/kg ITM	0 ±14.8	20 ±29.6	0 ±11.1	15 ±25.9	28 ±22.2	1 ml saline	50 ±29.6	70 ±29.6	45 ±37.0	NR	59 ±28.1
Shim #2 et al., 2021 [34]	0.20 mg ITM + 7.5 mg bupivacaine	22.4 ±8.14	35.8 ±17.8	NR		18.7 ±6.81	1000 mcg fentanyl + 90 mg ketorolac + 0.3 mg Naseron with 20 mcg fentanyl bolus and 5 mcg fentanyl basal infusion (IV-PCA)	29.6 ±14.0	49.4 ±17.0	NR		38.4 ±22.6
Chae et al., 2022 [28]	0.20 mg ITM + 1 mL saline	NR		NR		NR	0.5 ml saline	NR		NR		NR
Russo et al., 2022 [31]	0.15 mg ITM	NR		NR		0 ^*^	400 mg IV tramadol in 48 mL of 0.9% NaCl solution TAP block with 20 mL 0.2% ropivacaine	NR		NR		NR
Engström et al., 2023 [30]	0.10 mg ITM + 15 mg bupivacaine	NR		NR		NR	10 mg oral oxycodone	NR		NR		NR

Legend: ITM Intrathecal Morphine, IV-PCA Intravenous Patient Controlled Analgesia, NR not reported, TAP Transversus Abdominal Plane. Continuous data are presented as mean ± standard deviations unless stated otherwise. ^*^ Value represented as absolute values.

## Data Availability

All data are included in this manuscript.

## Supplementary Materials

The following supporting information can be downloaded at: https://www.mdpi.com/article/10.3390/jcm13010137/s1, Table S1: Secondary outcomes of included studies. Figure S1: Sensitivity analysis after exclusion studies not including basic analgesics in both groups. Figure S2: Funnel plot for publication bias in included studies.
