# Supplementary material for: Intrathecal Morphine and Post-Operative Pain Relief in Robotic Surgeries: A Systematic Review and Meta-Analysis
Source: J Clin Med. 2023 Dec 26;13(1):137. doi: 10.3390/jcm13010137 (PMC10779813; doi:10.3390/jcm13010137)

Supplementary Materials:

Table S1: Secondary outcomes of included studies

	ITM Group					Control						
**Author(s)**	**Nausea (%)**	**Vomiting (%)**	**Pruritus (%)**	**Urinary Retention (%)**	**Respiratory Depression (%)**	**Nausea (%)**	**Vomiting (%)**	**Pruritus (%)**	**Urinary Retention (%)**	**Respiratory Depression (%)**	**N/V prophylaxis**	**N/V treatment**
Segal et al., 2013 [32]	NR	NR	38 (n = 7)	NR	NR	NR	NR	0	NR	NR	4mg ondansetron	-
Bae et al., 2017 [27]	40 (n = 6)	20 (n = 3)	60 (n = 9)	NR	0	40 (n = 6)	6 (n = 1)	14 (n = 2)	NR	0	-	10mg metoclopramide
Koning et al., 2020 [9]	NR	NR	NR	NR	NR	NR	NR	NR	NR	NR	0.625mg IV dehydrobenzperidol + 4mg IV ondansetron	0.625mg IV dehydrobenzperidol
Shim #1 et al., 2020 [33]	30 (n = 9)	6.7 (n = 2)	16.7 (n = 5)	NR	NR	6.7 (n = 2)	0	0	NR	NR	0.0025mg/h basal infusion ramosetron	-
Dhawan et al., 2021 [29]	36.4 (n = 12)	0	21.2 (n = 7)	0	0	8.1 (n = 3)	0	5.4 (n = 2)	2.7 (n = 1)	0	-	Ondansetron
Shim #2 et al., 2021 [34]	24 (n = 6)	4 (n = 1)	16 (n = 4)	NR	0	8 (n = 2)	0	0	NR	0	0.0025mg/h basal infusion ramosetron	-
Chae et al., 2022 [28]	NR	NR	NR	NR	NR	NR	NR	NR	NR	NR	-	-
Russo et al., 2022 [31]	Recovery room: 18 (n = 2)<br>1H: 18 (n = 2)<br>12H: 9 (n = 1)<br>24H: 9 (n = 1)		0	NR	0	<u>TI group</u><br>Recovery room: 0<br>1H = 18 (n = 2)<br>12H = 27 (n = 3)<br>24H = 27 (n = 3)		0	NR	0	-	8mg ondansetron

						<u>TAP Block group</u> Recovery room: 9 (n = 1) 1H: 18 (n = 2) 12H: 18 (n = 2) 24H: 0						
Engström et al., 2023 [30]	NR	NR	NR	6.52 (n = 3)	0	NR	NR	NR	4.17 (n = 2)	NR	Betamethasone + ondansetron	-

Legend: NR not reported, TAP Transversus Abdominal Plane, TI Tramadol Infusion. Continuous data are presented as absolute values unless otherwise stated.

Figure S1: Sensitivity analysis after exclusion studies not including basic analgesics in both groups.

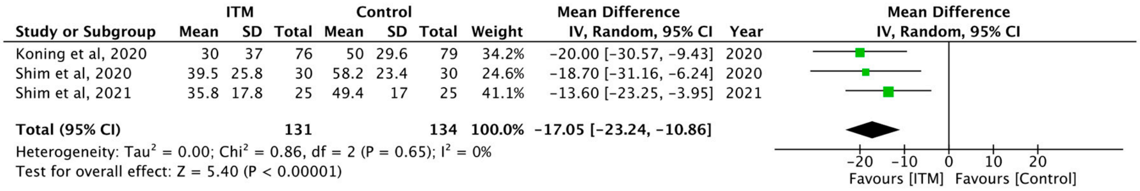

Figure S2: Funnel plot for publication bias in included studies.

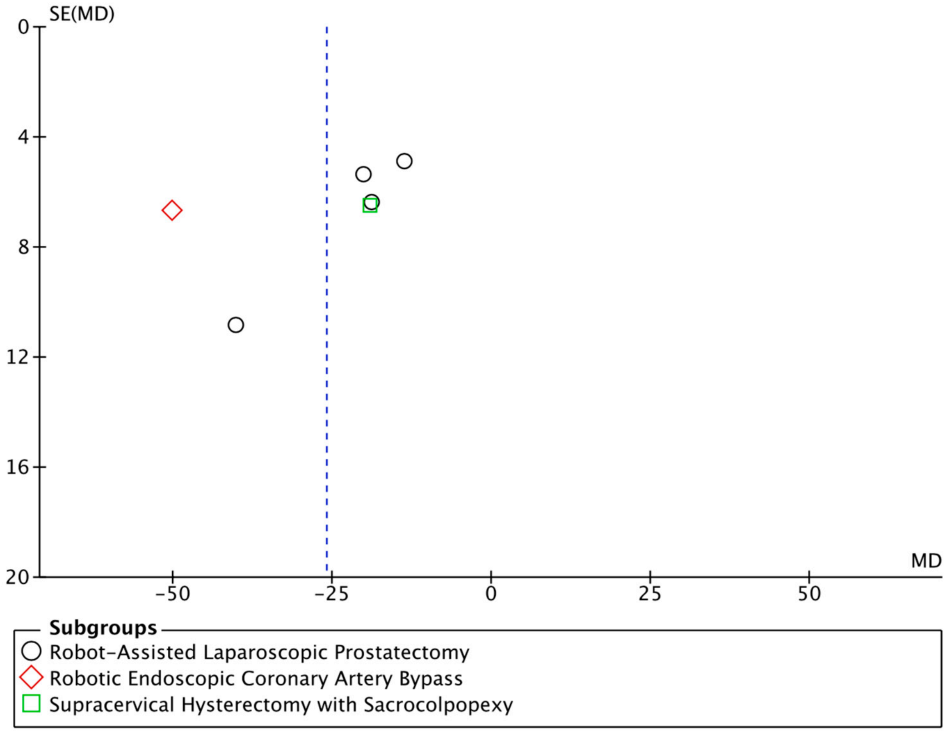

Supplement: Supplementary file 1 [file jcm-13-00137-s001.zip › jcm-2780772-supplementary.pdf]
